# Some Observations on the Prevention and Treatment of Hydrophobia

**Published:** 1791

**Authors:** William Loftie

**Affiliations:** Surgeon at Canterbury.


II. *Some Obfervations on the Prevention and Treatment of Hydrophobia. Communicated in a Letter to Dr.* Simmons *by Mr.* William Loftie, *Surgeon at Canterbury.*

ABOUT two years fince a poor man applied to me who had been bitten, both on the leg and arm, the day before, by a dog that died mad, after having bit two other dogs which were immediately killed.

The wounds were fmall; that on the arm was about two inches above the wrift, and only one of the dog's teeth had penetrated: the other, on the tibia, was more confiderable; here were the marks of two teeth.

In

In this caſe nothing was done till the time of his application to me, (twenty hours after the accident) when I diſſected out the bitten parts, taking off a piece of the integuments, of about the ſize of half a crown, and removing every part that had been even touched by the teeth.

Lint, dipped in a ſtrong ſolution of corroſive ſublimate, was applied to the parts, which were moiſtened at times with the ſame.

The next day, the arm and leg being much inflamed, an emollient cataplaſm was applied over the dreſſings, and the patient was directed to take ſome Glauber's ſalt.

On the third day the dreſſings were removed, a large eſchar was then formed, and there was a conſiderable diſcharge from the wound.

From this time the ſlough came off daily, and the wound diſcharged a laudable pus.

On the eighth day the patient complained that his mouth and gums were ſwelled; which muſt have been owing to abſorption of the corroſive ſublimate, as no mercurials were given internally.

The purging ſalt was occaſionally repeated, and the wounds were kept open for ſeven or eight weeks, when they were ſuffered to heal; and the patient has continued well ever ſince.

From

From every circumſtance I have no doubt that the dog by which this perſon was bit was mad; and I cannot help thinking that the patient's eſcape was entirely owing to the removal of the parts bitten, and keeping the wounds open ſo long.

Since that time another caſe of the ſame kind has fallen under my care, which I treated in a ſimilar manner and with equal ſucceſs; but in this the certainty of the animal being mad was not ſo clear.

Two caſes are related by Mr. Foot* of exciſion being attended with ſucceſs, in one of which the diſtance of time, between the bite of the dog and the extirpation of the part, was from thirty-two to thirty-five hours; and in the other, ſixty-eight hours. Such inſtances as theſe, and the firſt of thoſe which I have related from my own experience, evidently prove the uſefulneſs and neceſſity of extirpation; and indeed I have long been of opinion, from all I have heard and read on the ſubject, that nothing leſs than the complete exciſion of the parts bitten can be relied on, with any degree

* An Eſſay on the Bite of a Mad Dog. 8vo. London, 1788.

of of certainty, as a means of prevention in theſe unfortunate caſes.

In too many inſtances, however, either from the number and ſituation of the wounds, or from the fears of the patient, we ſhall be obliged, perhaps, to content ourſelves with deſtroying the parts bitten by cauſtic, which is certainly the beſt ſubſtitute for extirpation, though it has ſometimes failed of ſucceſs even in the ableſt hands *.

The caſes, by M. Sabatier, you have favoured me with from the Memoirs of the Royal Academy of Sciences at Paris for the year 1784, are ſtrongly in point, and confirm the neceſſity of deſtroying the wounded parts. With your leave I will copy them.—" On the 27th of

" February, 1784, a dog, kept by way of ſafe-

" guard in a lone houſe, bit the gardener be-

" longing to the houſe in the upper lip. The

" wound was dreſſed in the common way, and

" nothing more was done. The next day the

* See the caſe of Maſter Rowley, as communicated by John Hunter, Eſq., to Dr. Hamilton, and inſerted by the latter in his Remarks on the Means of obviating the fatal Effects of the Bite of a Mad Dog, &c. 8vo. London, 1785, page 211.

" ſame

" same dog, which had been shut up, but with-

" out being supposed to be mad, flew at a

" young man, who went to carry it some vic-

" tuals, and bit and scratched him in several

" places. The dog was immediately killed.

" Twenty-eight hours after the accident M.

" Sabatier applied liquid butter of antimony to

" the wounds, which were more or less consi-

" derable, and twenty-five in number, and to

" the scratches, of which he reckoned fifty.

" The most considerable were in each hand

" and fore arm, in the right shoulder, and in

" the left leg. These were kept open a consi-

" derable time, and the patient did well; but

" the gardener, who thought himself safe, and

" would not believe the dog had been mad,

" began, on the 14th of April, fifty-five days

" after the accident, to lose his appetite; the

" day following he complained of a pain in

" the wound, of a severe oppression at his sto-

" mach, and of a desire to vomit. The symp-

" toms of hydrophobia came on the same day.

" He was carried to the Hotel Dieu at Paris,

" and died on the 16th.

" M. Sabatier relates another case, which

" happened in 1775, of a soldier, bit also by a

" mad dog, where the caustic was applied

" with

"with fuccefs; while another man, who had "been bitten by the fame dog, was feized "with hydrophobia on the fifty-fecond day, "and died in twenty-four hours."

The method propofed by Dr. Haygarth, "of wafhing the wound with cold water, not "flightly and fuperficially, but abundantly, "and with the moft perfevering attention; in "bad cafes for hours; and after a plentiful "affufion of cold water, but not fooner, apply-"ing warm water *," is highly commendable for its fimplicity, and cannot be too much known and inculcated, as it promotes the wifhed-for defign, and gives time for medical affiftance.

The method you have been fo good as to communicate to me, as propofed by Profeffor Mederer, of Fribourg, and which confifts in wafhing the wound thoroughly, firft with a dilute folution of lunar cauftic in water, (in the proportion of thirty grains of the cauftic to a pint of water) and afterwards with warm water, feems alfo to be highly deferving of notice. At the end of the Profeffor's paper I obferve fome points of theory concerning the fuppofed action of fea water, in thefe cafes, from its al-

* See London Medical Journal, Vol. X. page 296.

kaline kaline contents, which may probably by some be deemed too fanciful; but this is a circumstance of no great consequence; and if you give the present remarks a place in the Medical Facts and Observations, I shall request you to add Professor Mederer's letter and other papers* on this subject, by way of note, or in any

* Cel. SIMMONS, Med. Londin. *S. D.* M. DE MEDERER, Professor Friburgensis.

De efficacia lixivii matricalis dilutissimi, ideo non amplius caustici, sed reagentis remedii contra hydrophobiam prophilactici persuasus nuper in litteris ad te datis rogavi, qui, si quis a bestia rabida demorsus se curandum sisteret, illum methodo in syntagmate de rabie canina a me descripta tractare velis ut sic & usu confirmaretur, quod ratione demonstratum est; nam cum id rogarem, propriis experimentis certis, quibus theoriam meam comprobarem, adhuc destitutus fui. Verum non diu post accidit, ut duæ a fele rabida demorsæ ancillæ, & nuper denuo, ut tres a cane rabido admorsæ personæ præfata mea methodo curarentur, & omnes feliciter conservarentur; quorum factorum speciem simulque læsionis & sanationis historiam ex testimoniis authenticis in hunc finem annexis colligere licet.

Cum verba tantum moveant, exempla vero trahant, jam nunc futurum existimo, ut Philiatrorum nemo amplius dubitet, data quavis occasione pharmacum non causticum (nam lapidis caustici grana triginta in aquæ libra una soluta non amplius adurunt) nihilominus tamen contra hydrophobiam prophilacticum adhibere, cujus efficacia non solum a priori, scilicet any other form you pleafe, as I am perfuaded there are many readers who, like myfelf, will be glad to have a copy of them.

That fcilicet ratione & analogia demonftrata, fed & a pofteriori ipfa nempe experientia confirmata fuit. Quod ut omnes ac finguli quacunque artis faluberrimæ praxi occupati faciant, valde exopto, quo multiplicato demum experimentorum numero omne dubium evanefcere, atque fic tandem fieri poffit, ut non folum calamitas hæc a miferis repellatur, fed & ceteri ea moleftia liberentur, quam aliena miferia affert. Nullus præterea dubito, quemquam fore, qui æque opportunam quam certam hanc curandi methodum non omni alii magis profecto ambiguæ & incommodæ præferat. Nullum enim aliud remedium prophilacticum certius hucufque inventum effe, ex eo facile arguitur, quod non ita pridem adhuc a beftiis rabidis admorfi rabie implicarentur, & ea oppreffi, licet a peritiffimis in arte viris & juxta normas noviffime præfcriptas curarentur, nihilominus tamen infeliciffime perierint plurimi; mea vero methodo tractati confervati fuerint omnes.

Cum tefte Celfo & omnibus, qui miferos fitire & rabire viderunt, hydrophobia cum rabie non folum miferrimum morborum genus, fed etiam eo oppreffi in maximo difcrimine fint, ab hac calamitate & periculo homines confervando hominibus maximum & diftinctiffimum officium præftari, & ideo neminem, qui officium hoc præftitit, maxima voluptate non affici neceffe eft, quare omnes, quibus fortuna, aliquem hac mea methodo ab ifta calamitate confervare contigit, ut mihi eam fignificare, & fic voluptatem fuam ob hominem confervatum communicare non graventur, etiam atque etiam rogo. Vale.

Dabam Friburgi Brifgoviæ die 1ma Sept. 1789.

*Copia*

That persons sometimes escape the effects of the bite of rabid animals, without any prophylactic

*Copia Attestati supremæ præfecturæ Serenissimi S. R. I. Principis a* Fürstenberg *in valle Kinzingana.*

In ditione Serenissimi Principis a Fürstenberg & quidem in supremæ præfecturæ vallis Kinzinganæ dynastia Wolfach dicta, rusticus Andreas Ehle, subditus in Rankach 13tia Septembris hujus anni denunciavit, duas prædii sui ancillas Agatham Müllerin unam, & Mariam Annam Borhoin alteram a fele rabiosa demorsas fuisse.

Mandabatur illico provinciæ physico & chirurgo, ut necessaria interim remedia adhiberent, sed & eodem tempore ad D. Professorem Mederer Friburgum tabellarius missus est, qui eundem rogaret, ut suum contra hydrophobiam laudatum remedium mittere, modumque applicandi edicere, vel si non gravaretur, ipsus venire vellet.

Venit ipse ea, qua ob longam distantiam licuit, celeritate; ancillas morsu offensas comitante Domino Consiliario intimo & supremo Præfecto, pone insequentibus D. dynastiæ Consiliario, D. Physico & Chirurgo invisit, vulnera inspexit, suo remedio lavit, deligavit, addito simul mandato, ut hæc curandi methodus iterato repetatur, nullum præter hoc aliud remedium applicetur, postea vero vulnera consuetis sanandi regulis curentur.

Per longius deinde tempus hanc ob caussam hic loci commoratus prædictus D. Professor de læsarum ancillarum circumstantiis a loci chirurgo indies edoctus, easdemque cum ante abitum adhuc visitaret, sanatas fere reliquit, domumque suam repetit.

lactic means being employed, is certain, and must be accounted for from some particular state

Nos nunc ex integro sanatas curatasque esse cernimus, quod unice sanandi methodo prædicti D. Professoris in acceptis referendum esse putamus.

In cujus rei testimonium patentes hasce litteras majori supremæ Præfecturæ nostræ sigillo munitas dedimus.

*Wolfachi* die 8va Decembris 1783.

(L. S.) Cancellaria supremæ Præfecturæ Serenissimi Principis a Fürstenberg in valle Kinzingana.

*Copia Attestati D. provinciæ Physici D.* Wegbecher.

Infra scriptus a supremo huiate Præfecto die 13tia Septembris h. a. mandatum accepi, ut me cum chirurgo Rankachium dynastiæ Wolfacensis vallem conferrem, ibidemque duarum a fele rabida demorsarum ancillarum curam gererem. Verum cum mihi notum esset, D. Mederer Professorem Friburgensem certum contra rabiem habere remedium prophilacticum, rogabam amplissimam Præfecturam supremam, ut præfato D. Professori casum hunc nuntiaret, simulque ab eo requireret, ut vel ipse venire, vel remedium suum cum instructione, quomodo applicari debeat, mittere velit.

Adveniens in Rankach intellexi, felem, quæ ambas ancillas momorderat, tribus diebus absentem fuisse, nempe non, ut hucusque solebat, mane & vespere, dum vacæ mulgebantur, ad stabulum venisse, ubi ab ancillis prædictis semper lacte pascebatur. Quarta autem die mane iterum ante domum comparuisse, & in ibi consistentes ancillas irruisse, unius pedem

ſtate of the habit at the time. We daily ſee inſtances where inoculation fails, though the insertion dem lædens; inde verberibus deterrita alteram petiit, pedem primo, ab hoc vi depulſa ejuſdem ancillæ manum mordens; tandem in terram depreſſam felem a ruſtico ad ancillarum clamorem advolante furca interfectam fuiſſe.

His in adventu meo auditis juſſi, ut felis necata aperiretur, apertamque inſpiciens inveni, linguam aridam, tumidam & uſquequaque nigram, œſophagum vero & aſperam arteriam cum maxima parte pulmonum inflammatum.

Ex his & ex factis præmiſſis judicabam, felem revera rabioſam fuiſſe, ideoque morſuum vulnera dilatari, muria lavari & unguento digeſtivo obligari juſſi, quod & illico factum fuit.

Quamprimum D. Profeſſor Mederer advenit, ego & ipſe cum ſupremo Præfecto D. Schwab, cum præfecturæ Conſiliario D. Battie & Chirurgo D. Schroff inviſebamus admorſas, quæ de fato ſuo ſollicitæ & triſtes, clavi S. Huberti in vola manus aduſtæ, a D. Profeſſore bono animo eſſe jubebantur, auxilium ipſis infallibile promittendo.—Fidebant miſeræ promiſſis eo certius quod anno vix dum præterito undecim perſonas morſu rabioſi canis læſas ab eodem D. Profeſſore ſanatas fuiſſe atteſtabamur.

Aperiebantur earum vulnera obligata, lixivio, quod ex granis triginta lapidis cauſtici chirurgorum & libra una aquæ communis paratum fuit, diligenter eluebantur, & carpto mox dicto lixiviæ madefacto denuo obligabantur, quæ operatio ut una alterave vice repeteretur, juſſit, aliud quodvis remedium applicari insertion of the variolous matter is carefully done, and other patients inoculated at the same time, applicari prohibuit, vulnera vero methodo vulgari curanda præcepit, quod accurate factum fuit.

Vigesima tertia Septembris denuo invisebamus ego, D. Professor Mederer, D. Dr. Metzler Comitis Bissing Medicus & Consiliarius, D. Consiliarius Battie & D. Chirurgus Schroff demorsas, & illas sortis suæ certiores solatii plenas invenimus. —Vulnera erant moderate inflammata, optimam suppurationis speciem prodentia; in manu vero ancillæ alterius detegebamus, morsum, quem prius tantum superficialem credebamus, per cutem penetrasse; dilatari itaque & hoc vulnus — prædicto lixivio elui, & eo, quo prius modo obligari præceptum est.

Nunc post septimanas circiter quinque vulnera omnia sine ullo malo symptomate sanata, & ancillæ sanæ & alacres esse deprehenduntur, quod methodo rationali D. Professoris attribuo. In cujus testimonium hasce litteras proprio sigillo munitas scripsi.

*Wolfachi* 6ta Decembris 1783.

(L. S.) WEGBECHER Medicinæ Doctor, Serenissimi Principis a Fürstenberg Consiliarius, & dynastiarum Wolfach & Haslach Physicus.

Præpositum attestatum propria manu serenissimi Principis a Fürstenberg Consiliarii — Med. Doctoris & supremæ præfecturæ in valle Kinzingana Physici ordinarii, D. Jacobi Wegbecher time, with the ſame matter, and the ſame lancet, have the diſorder. May not the ſame thing happen becher hic Wolfachi ſcriptum fuiſſe appreſſo cancellariæ minori ſigillo atteſtatur

*Wolfachi* 8va Decembris 1783.

(L. S.) Cancellaria ſupremæ præfecturæ Sereniſſimi Principis a Fürſtenberg in valle Kinzingana.

*Copia Atteſtati Chirurgi jurati D.* Schroff.

Infra ſcriptus per ſupremam præfecturam cum D. Phyſico D. Wegbecher ad duas illas a fele rabioſa in Rankach demorſas ancillas miſſus fui, quas læſas inveni, ut ſequitur.

Agatha Müllerin 18 annorum, in crure dextro, parte media anteriori duplicem excoriationem, parte exteriori vero & ſuperiori morſum duplicem per cutem profunde in carnem ſuræ penetrantem paſſa eſt.

Maria Anna Borhoin 22 annorum in parte poſteriori & media ſuræ ſiniſtræ morſum unum duplicem per cutem profunde in carnem penetrantem — in parte exteriori brachii ſiniſtri proxima ad carpum morſum alium duplicem per cutem profunde penetrantem accepit; præterea in carpo ipſo morſus, qui primum excoriatio tantum videbatur, poſt ſuppurationem vero profunde ſub cute penetraſſe inventus eſt.

Singula cutem penetrantia vulnera a me dilatabantur, morſus duplices penitus conſcindebantur — prima vice tantum ſecundum ordinationem D. Doctoris Wegbecher, poſtea vero ſemper ſecundum methodum D. Profeſſoris Mederer curabantur, cui ſoli adſcribo, quod demorſæ ſeptimana quinta, ſine

 ullo happen in the bite of a mad dog? — I remember a case of a child at the breast, being for a fortnight ullo malo symptomate sanatæ fuerint, & adhuc omnimode sanæ & alacres sint.

Quod propria manu sigilloque attestor *Wolfachi* 6ta Decembris 1783.

CONRADUS SCHROFF, chirurgus juratus.

(L. S.)

Præpositum Attestatum propria manu huiatis chirurgi jurati Conradi Schroff scriptum esse, appresso minori sigillo testatur *Wolfachi* 8va Decembris 1783.

(L. S.) Cancellaria supremæ præfecturæ Serenissimi Principis a Fürstenberg in valle Kinzingana.

Nos ad supremam præfecturam vallis Kinzinganæ constituti serenissimi Principis a Fürstenberg Consiliarius intimus & Præfectus supremus nec non Consiliarii & officiales attestamur præsentibus, duas illas ancillas in rustici Andreæ Ehle Domu in valle Rankach præfecturæ Oberwolfach decima tertia Septembris 1783, a fele rabida demorsas, scilicet Annam Mariam Borhoin ex præfectura Oberwolfach & Agatham Müllerin ex valle imperiali Hammerspach methodo a Friburgensi D. Doctore & Professore Mederer proposita feliciter sanatas, hucusque semper sanas & alacres fuisse, & adhuc optime valere.

*Wolfachi* die 20 Decembris 1784.

(L. S.)

Copia fortnight or more in a room where four or five children had the ſmall pox, and ſome of them the

*Copia Atteſtati D. D. Conſiliar. aulic. & Medic. D.* Poſch *& Chirurgi* Panck.

David Mayer murarius triginta trium annorum, Bubſhemii in cæſ reg. A. A. dynaſtia Hochenberg natus, 24ta Julii anni currentis in via Hüfinga Doneſchingam ducente a cane rabido per togam ex cannabe textam & induſium in cubitum dextrum ita admorſus fuit, ut vulnus rotundum quadrantem pollicis latum & profundum in muſculum extenſorem brevem cubiti prope flexuram ſurſum verſus penetraret.

Franciſcus Xaverius Hefler undecim annorum & trium menſium filius civis Doneſchingenſis ab eodem cane, a quo David Mayer vulneratus fuit, admordebatur in pede dextro duos pollices ſupra condilum externum. Hoc morſu duo infligebantur vulnera, primum æque rotundum quadrantem pollicis latum, medium vero profundum intrabat ſupra condilum externum inter muſculum peroneum anteriorem & poſteriorem, os tamen peroſtio [illegible]o adhuc tectum fuit. Secundum vero duos pollices a priori diſtans ſuper tibiam in tibiæo antico ejuſdem latitudinis, non vero tantæ profunditatis erat.

Ab hoc puero canis rabidus inſiliebat in puellam 9½ annorum, hujatis militis filiam, Franciſcam Roeſch, & proſternebat illam retrorſum in terram. Canis hanc puellam admordebat in brachio dextro ad articulum brachii anterioris cum ſuperiore, duoque vulnera infligebat, unum ſuper cubitum in muſculum extenſorem brevem, alterum ſub cubito introrſum in muſculum cubitæum internum, utrumque quadrantem pollicis latum & profundum. Simul in ejuſdem brachii media &

the confluent fort, without catching the difeafe, though, in general, it is fo eafily contracted.

When

& interna parte, ubi caro mufculi radiæi interni in partem fuam tendinofam definit, per cutem nudam fauciabatur vulnere fabæ italicæ magnitudine, & $\frac{1}{4}$ pollicis profunditate. Præterea eidem puellæ idem canis rabiofus morfu inferebat vulnus in labium oris fuperius verfus finiftram lentis vulgaris magnitudine.

Poft hæc facta nos, ut in rei veritatem accuratiffime inquiriremus, & miferis auxilium efficaciffimum afferemus, illico appellati fuimus.

Inveniebamus tunc has tres admorfas perfonas fine ullo adverfo fymptomate.—Vulnera igitur illico dilatabantur, & poftquam fanguis ad fufficientem quantitatem efluxiffet, illa lixivio, quod ex lapide cauftico gr. XXX & fufficienti aquæ quantitate libr. I. paratum fuit, fuadente D. Matthæo de Mederer, &c. &c. eluebantur, nec non carpto eodem lixivio madefacto deligabantur, & fecundum artis principia curabantur. Vulnera hoc lixivio per Menfem in fuppuratione confervabamus, & toto hoc curationis tempore nullum adverfum fymptoma obfervabamus.

Elapfo menfe vulnera jam penitus erant fanata, & hæ tres perfonæ hucufque adhuc optime valent. Quamobrem hujus beneficii nunquam non memores Domino Inventori præftantiffimi hujus remedii ob ejus divulgationem gratias referimus debitas, & hifce facta hæc palam conteftamur,

Atteftatum præpofitum ab huiate Sereniffimi Principis Confiliario aulico & medico D. Pofch & ab huiate Sereniffimi Principis Chirurgo de Panck manu propria fubfcriptum &

Confueto

When the means of prevention have been neglected, or have failed, and hydrophobia has actually

Conſueto illorum ſigillo munitum, ſimilibus vero ab his exhibitis Atteſtatis omnimode credendum eſſe, ſub ſigillo majori Cancellariæ Regiminis Sereniſſimi Principis a Fürſtenberg & ejuſdem Secretarii Nomine manu propria ſubſcripto hiſce in optima forma affirmatur.

*Doneſchingæ* 23 Decembris 1784.

Sereniſſimi Principis a Fürſtenberg

(L. S.) Regimen hujas.

FREY Conſil. & Secretar.

*Copia Atteſtati Regiminis Sereniſſimi Principis a* Fürſtenberg.

Nos ad Regimen Sereniſſimi Principis a Fürſtenberg conſtituti Præſes, Cancellarius, Conſiliarii intimi & aulici atteſtamur hiſce—die 23tia Julii anni currentis nobis durante ſeſſione a politiæ miniſtris annunciatum fuiſſe, quod canis rabidus in oppidum irruiſſet, & homines & pecora læſiſſet, nec non quod jam diſpoſitio neceſſaria facta eſſet partim, ut homines læſi convenienter curarentur, partim ut canis rabidus deprehenderetur & interficeretur, nec non ut pecora læſa majoris ſecuritatis cauſſa illico necarentur, & terra abſconderentur.

Ex continuo facta inveſtigatione ſequentia inventa fuere. Canis prædictus erat alienus, hic loci ignotus, fuſcus, villoſus, venaticus, armilla coreacea aurichalco præfixa cinctus; veniebat ſuper viam publicam Huffinga ad oppidum noſtrum hora media undecima ante meridiem. Adhuc in diſtantia 200 paſſuum ante oppidum inſperato & non irritatus irruebat in ſuper viam mox dictam ambulantem Davidem Mayer murarium actually taken place, what are the moſt proper remedies to be adopted? The antiſpaſmodic and rium ex Bubſheim atque inferebat ipſi vulnus in cubitum dextrum. Ab hoc ſecum portato tigni fruſto repulſus canis prædictus capite ſemper demiſſo, lingua exſerta, ore ſpumante, & cauda retracta directe ſe in oppidum noſtrum conferebat, & ſtatim ad primam domum canem ad eam in platea ambulantem opprimebat. In oppidum ingreſſus lacerabat gallinam ipſi obviam, & intrabat in domum apertam & ejus conclave inferius, in quo bona fortuna neminem inveniebat. Ex hac ſe in partem exteriorem hujus loci recipiebat, & adgrediebatur Franciſcum Hefler, puerum undecim annorum ſub foribus paternæ domus nudis pedibus ſtantem, & in ejus pedem ſiniſtrum dentes adeo defigebat, ut affixus pedi a puero ultra tres paſſus in domum retraheretur, ubi a patre & fratre majore advolantibus fuſtibus dejiciebatur.

Vix vero 50 paſſus abhinc invadebat a tergo Franciſcam Roeſchin, puellam novem annorum in platea exiſtentem, proſternebatque eam in terram, & morſu ſauciabat brachium ejus dextrum, & labium oris ſuperius.

Tunc præciſe ſubulcus gregem ſuam domum cogebat. Prædictus canis rabidus iterato in eam penetrabat, & tres porcos vulnerabat. Poſtea ex loco ſupra viam Schweningam ducentem pedes efferebat; ante ultimam domum vero adhuc canem & felem per plateam incedentem aggrediebatur & vulnerabat. Tandem extra oppidum circiter 150 paſſus procumbebat ad viam publicam in foſſa ſicca & ceſpite obducta, ubi a Principis chirurgo D. de Panck glande in maxillam inferiorem ictus fuit.

Hoc

2

and nervous medicines, which are so generally had recourse to in these cases, have so often failed

Hoc ictu vulneratus canis alia via se denuo in oppidum recipiebat, & statim primo ingressu allata agressus, gallum & gallinam sauciabat.—Ultro in locum irrupturus, a venatore Carolo Goenner secundum glandis ictum in ventrem accipiebat, accepto se in stabulum ex adverso apertum, & per hoc in domum ipsam recipiebat, ubi reclusus a Cels. Regiminis scriba Scheidegg primo punctibus vulnerabatur, & tandem glandis ictu trajiciebatur. Canis hic nunquam, nec verberatus, nec punctus aut ictus vocem edidit.

De personis vero sauciatis referebat Consiliarius aulicus & principis medicus ordinarius D. Posch, nec non principis chirurgus D. de Panck, quorum curæ traditæ fueræ, quod prædictæ personæ methodo D. Professoris Friburgensis de Mederer tam fortunato successu tractatæ fuerint, ut non solum tempore curæ semper alacres, sed etiam quatuor post septimanas jam penitus sanatæ, & hac cura ab omni periculo rabiei immunes redditæ fuerint.

Sicut & hæ personæ post decursum 5 mensium adhuc optime valent.

Ad tuendam veritatem & majorem confirmationem prædictorum hoc Attestatum majori nobis commisso Regiminis Insigni corrobari, & consueto more per Secretarium nostrum subscribi jussimus. Quod factum est *Doneschingæ* die 23 Decembris 1784.

Serenissimi Principis a Fürstenberg Regimen hujas.

(L. S.)

FREY Consil. & Secretar.

*Methodus*

failed of ſucceſs, that I have long determined, in my own mind, ſhould any inſtance of this dreadful

*Methodus facillima & certiſſima Homines & Animalia cuncta a Beſtiis rabioſis admorſa conſervandi, ne quoque in rabiem deveniant.*

§. 1. In curatione hominis a beſtia rabida (cane aut felle) demorſi id imprimis agendum eſt, ut virus vulneri immiſſum deſtruatur, priuſquam abſorptum atque univerſæ humorum maſſæ commixtum fuerit. Hoc virus per ſeptimanas, menſes loco, cui adplicatum fuit, iners bona fortuna hæret.

§. 2. Quo fine exſciſſio aut inuſtio vulneris probatiſſimum eſt & princeps remedium a Celſo jam recommendatum; quomodo hæc illave inſtitui debeat, cuilibet Chirurgum notum erit.

§. 3. Verum, cum hæc medendi methodus, quia crudelis adparet, ſæpe repudietur, impoſſibilis ſæpe ſit, his in caſibus ſecuturæ rabiei periculum perſiſtit, niſi alia iſthæc ratione avertatur.

§. 4. Cum experientia certo conſtet, ex remediis omnibus hactenus eo ſcopo laudatis nullum infallibile fuiſſe, aliorum auxiliorum tentamen nemini abſonum videbitur, præſertim ſi eorum efficacia prævideri poſſit, & obſervationibus jam comperta fides ſit. Quale remedium eſſe videtur illud a Profeſſore Friburgenſi de Mederer nuper publici juris factum, lixivium nempe matricale ita dilutum, ut non amplius adurat. Methodus illud adhibendi ſequens eſt.

§. 5. Quodlibet vulnus ab animali rabido aut de rabie tantum ſuſpecto admorſum omnium primo, ſi anguſtum & profundum ſimul fuerit, lege artis dilatetur, tum lixivio ſupradicto dreadful diforder fall under my care, to change the method of treatment, and try the effects of tonics.

The dicto (§. 4) eluatur, (ex granis triginta lapidis cauftici Chirurgorum & libra una aquæ ex tempore parato,) fi locus non nimis fenfilis illud permittat, carptis eodem ebriis deligetur, fi vero locus admodum fenfilis effet, lixivio mox dicto probe abftergatur, dein aqua communi tepida rurfus eluatur, ac denique ligaminibus ficcis obligetur.

§. 6. Deterfio lixivii ope aliquoties per diem iteretur, tam diu, quam per inflammationem licuerit.

§. 7. Si vulnere jam inflammato vocaretur Chirurgus, expectanda ei fuppuratio eft, atque tum methodo fupra defcripta (§. 5) ulcus tractandum.

§. 8. Si ferius adhuc, vulnere nimirum pro parte, aut integrum jam fanato accerferetur, illud lapide cauftico denuo exulcerare, atque ulcus poft deciduam Efcaram lixivio fæpius memorato eluere & deligare debet. Non perinde eft hic, lapis caufticus aliudve caufticum recipiatur, ille enim partes animales & cum his virus rabiofum multo certius deftruit, quam quodcumque aliud caufticum ex vitriolorum profapia.

§. 9. Vulnera quæcumque ex dictis (§. 3 vel 5) methodis tractata cæterum juxta generales artis leges fanantur.

§. 10. Cum hac ratione virus rabiofum admorfo in loco deleatur, atque ideo nil de eo reforberi poffit, omnia remedia interna, externaque ad veneni reforptionem impediendam, illudve jam reforptum deftruendum hactenus laudata plane fuperflua funt.

§. 11.

The plan I have propofed to myfelf would be, (after fuch previous evacuation as might feem

§. 11. Omnes (§. 3 vel 5) dictis methodis tractati rabie corripi omnino nequeunt. Quod fi vero omiffionis caufa accideret, in horum infortunatorum hominum cura nullum philantropiæ officium prætermittatur, hoc eo magis fieri poteft, cum convicti modo fimus, ejufmodi infortunatos homines non mordere, & falivam fine morfu non inficere.

§ 12. Verum non quilibet admorfus, qui ex angore, ne rabidis moriatur, moeftus eft & meticulofus, eadem ex caufa varii generis fymptomatibus, hydrophobiæ analogis urgetur, pro rabido ftatim declarandus eft. Inde accidit, quod tam diverfis fæpe fibi contrariis remediis adeo multi a rabie fanati legantur. Anxiis his folatium præbeatur, & fi dicta (§. 5 = 8) methodo nondum tractati funt, eadem tractentur.

§. 13. Vera rabies communiter inter tres feptimanas totidemque menfes erumpit; quod de multo citiore vel multo tardiore ejus eruptione fcriptum habetur, id incertum eft.

§. 14. A prægreffo vehementi corporis, animive motu ut plurimum excitatur, vulnus adhuc apertum vel jam claufum de novo dolere incipit, dolores centrum corporis gradatim petunt, plerofque alternum frigus adoritur cum laffitudine, febris fymptomatibus confuetis plus minufve ftipata, his fe affociat deglutiendi impotentia (unde perpetua illa fputatio) ac infuperabilis denique horror non ab omni folum liquido, verum & eo omni, quod illius ideam excitare valet.

§. 15. Utrumque hoc fymptoma *Difphagia* & *Hydrophobia* effentiales rabiei characteres conftituunt, ejus præfentiam illa unice definiunt.

§. 16.

ſeem neceſſary) to direct my patient to be kept as much from the light as poſſible; the bark, in ſubſtance, to be given in large quantities, and Port wine plentifully; but that every thing liquid ſhould be given from a dark-coloured, unglazed tea pot, that nothing might appear to the patient; that bark clyſters, with opium, ſhould be frequently thrown up; oil of amber

§. 16. Miſerrimum hoc morborum genus arti medicæ indomabile adhuc eſt, & cum in mox dictis (§. 15) ſymptomatibus principaliter illud conſiſtat, interna medicamenta incaſſum hæc quærit. Externa quærenda ſunt remedia, inunctio mercurialis hactenus laudata in rabie jam præſente manifeſto nociva fuit*; balneum ex aqua marina ſemper profuiſſe legitur †; profuiſſe poteſt, quia ex aqua balneo adhibita reſorberi quid & lymphæ, proprio virus rabioſi vehiculo admiſceri poteſt.

§. 17. Si balneum maris hoc in caſu ſemel juvit, id alcali ex aqua marina reſorpto certe debetur; an lixivium dilutum huic fini non imprimis utile foret? In virus ſcrophuloſum potenter agit, veneno rabioſo plus quam venereum adfine, quocum poſteriore Sauvageus tantam veneni rabidi analogiam reperiit.

§. 18. Hinc actu rabidi hydrophobia non obſtante in balneum lixivioſum provide demittendi, atque in eo tamdiu detinendi eſſent, quam fieri poteſt; *nam in deſperatis anceps remedium experiri melius eſſe, quam nullam,* Celſus jam profeſſus eſt.

* Moreau. † Tulpius.

 rubbed rubbed on the vertebræ of the neck and back, and veficatories, as ftimulants, applied to the throat; and that, as foon as poffible, the cold bath, or, what may be more eafily ufed, the fhower bath, fhould be had recourfe to.

I have faid nothing of the ufe of mercury, which has been fo often recommended, and found inefficacious in thefe melancholy cafes, becaufe the action of it, if there be time for it to enter the circulation, feems likely to counteract the bark and the other tonic remedies I fhould wifh to employ.

I have been confirmed in my opinion of the propriety of at leaft making trial of the mode of treatment I have ventured to fuggeft, by reading fome obfervations on the caufe and cure of the tetanus, lately publifhed by Dr. Rufh, of Philadelphia *, in which he gives an account of the fuccefs of the bark and wine, taken in large quantities, in that difeafe. To thefe, in one cafe, he added a blifter between the fhoulders, and, in another, the oil of amber in large dofes, when he fufpected the bark and wine began to lofe their effect.

* See the London Medical Journal, Vol. VII. page 424.

After

After aſſigning his reaſons for throwing aſide opium and nervous medicines, he proceeds to obſerve, that, having had no opportunity of ſeeing the hydrophobia ſince he had adopted theſe principles, he is unable to determine how far his reaſoning with reſpect to tetanus may be applicable to the hydrophobia; but from the ſpaſmodic nature of the latter diſorder, from the ſeaſon of the year in which it generally occurs, and, above all, from a caſe related by the late Dr. Fothergill, of a young woman having eſcaped the effects of a mad cat by means of the wound being kept open, (and which, from its ſeverity, Dr. Ruſh thinks was probably connected with ſome degree of inflammation) he aſks whether it is not probable that the ſame remedies, which have been employed with ſucceſs in the tetanus, may be uſed with advantage in the hydrophobia?

At the concluſion of his paper he very properly remarks, (and the obſervation may ſerve as an excuſe for the hints which I myſelf have ventured to throw out on the ſubject) that in a diſeaſe ſo deplorable, and hitherto ſo unſucceſsfully treated, even a conjecture may lead to uſeful experiments and inquiries.

Although the caufe of tetanus, and of hydrophobia, may have a different origin, yet the effect in both feems, in fome manner, to agree, a fpafmodic affection of the mufcles, and particularly of thofe belonging to deglutition, being brought on. I am willing to allow that the nervous fyftem is likewife affected, as the difeafe is always increafed upon even the approach of liquids, without attempting to drink, or by any thing of a fhining nature.

Before I conclude this letter I fhall take the liberty of offering a few more remarks relative to this fubject, which have been fuggefted by a perufal of Dr. Percival's hints towards invefligating the nature, caufes, &c. of the rabies canina, addreffed to Dr. Haygarth, and inferted in the tenth volume of the London Medical Journal. The learned author fays he does not perceive any ftrict analogy between the action of the canine virus and that of lues venerea, fmall pox, or of the viper; as thefe evidently affect the lymphatic fyftem, and their progrefs into the courfe of circulation may be readily traced, which is not the cafe with the bite of a mad dog.—" Are we then," he afks, " funda-" mentally right in the idea, that the bite of a " rabid animal operates by abforption ? and

" might

" might not its effects be, at leaft as well, if not " better, explained, by afcribing them to local " nervous irritation, propagated at different pe- " riods of time, according to the varying cir- " cumftances of fenfibility and irritability to " the brain, and from thence to the fauces, " gullet, and ftomach ?"—This doctrine is ingenious, but not fo clear, I apprehend, as that of abforption. It is true there is a great difference between the virus of rabid animals and of thofe diforders which the Doctor mentions, but yet the affection may be eafily accounted for by abforption, if we allow that a greater length of time is required for the action of the one than is neceffary for the other. We know that in the fmall pox and lues venerea the infection is found to have taken place in a few days, and in fome cafes in a few hours; in the more active contagion of diforders, from putrid affection by infertion, in a very fhort time indeed. About thirty years fince I accidentally wounded my finger with the point of my knife in opening a woman who died of a dropfy of the ovarium, where the contained fluid was very putrid. In a very fhort time I felt a flight uneafinefs or irritation in the part, and in the courfe of the night it might be traced to the glands above the elbow, and from thence to the axilla, where a collection of matter formed, which I am inclined to think saved my life; and the morning following the accident a ripe pustule was observed on the punctured part.

The virus of rabid animals will certainly lie dormant for weeks, till some change takes place in the habit, when it becomes active. — Dr. Hamilton, in his remarks on this subject, (pages 99 and 108) says, that the time required for the virus to become active is rather uncertain; but he thinks from four weeks to three months are by much the most frequent, and that the first symptom is generally a pain in the part where the bite has been received, stretching in the course of the lymphatics towards the heart, or where they unite with the sanguiferous system.

Mr. Jesse Foot* thinks that "forty days is "about the general average from the bite to "the time of the coming on of hydrophobic "symptoms;" though there are cases on record where the morbid affection has not shewn itself for some months, even to the eleventh, and,

* Essay on the Bite of Mad Dog.

as in the cafe related by Mr. Nourfe †, to the nineteenth. Still, however, in general, the appearances take place in the bitten part firft, and from thence are conveyed by the lymphatics, or fome other feries of veffels, to the circulation. It is uncertain what ftimulates the virus to action, but the effect feems to me to be the fame as that caufed by inoculation, &c.; a local irritation is brought on, and thence communicated to the habit.

Dr. Percival, in the paper already referred to, fays, "the acceffion of canine madnefs is "uncertain as to the diftance of time from the "bite, and the fymptoms by which it firft "manifefts itfelf: but frequently the cicatrix "becomes hard and elevated; pains fhoot from "it towards the head; it is furrounded with "livid or red ftreaks, and the wound breaks "out afrefh." This is coming very near the action of the venereal difeafe or fmall pox, though the time is fo uncertain. It is particu-

† Philofophical Tranfactions, Vol. XL. page 5. See alfo a cafe of hydrophobia related by Mr. Dundas in the London Medical Journal, Vol. VIII. page 156, where eighteen months intervened between the bite and the acceffion of hydrophobia.

larly happy for mankind that the virus of rabid animals requires ſo much time to vegetate, (if I may be allowed the expreſſion) as it admits of preventives to be made uſe of, and none, as I have already remarked, ſeems ſo likely to ſucceed as exciſion.

*Canterbury,*
January 24, 1791.

